# Cancer-associated fibroblast-mediated immune evasion: molecular mechanisms of stromal-immune crosstalk in the tumor microenvironment

**DOI:** 10.3389/fimmu.2025.1617662

**Published:** 2025-08-26

**Authors:** Junling Luo, Xuehua Xiang, Guangyuan Gong, Lang Jiang

**Affiliations:** ^1^ Department of Medical Laboratory, Jiangsu Provincial People’s Hospital Chongqing Hospital, Chongqing, China; ^2^ Department of Medical Laboratory, Qijiang District People’s Hospital, Chongqing, China; ^3^ Department of Public Health, Jiangsu Provincial People’s Hospital Chongqing Hospital, Chongqing, China; ^4^ Department of Intensive Care Medicine, Jiangsu Provincial People’s Hospital Chongqing Hospital, Chongqing, China

**Keywords:** cancer-associated fibroblasts, immune evasion, immune cells, tumor microenvironment, CAFs

## Abstract

The tumor microenvironment (TME) is a complex ecosystem and cancer-associated fibroblasts (CAFs) are critical drivers of the immunosuppressive TME. The dynamic interactions between CAFs and immune cells play a crucial role in tumor progression and immune evasion. This review systematically investigates the interactions between CAFs and different immune cells and elaborates on the molecular mechanisms of CAF-mediated immune suppression, with a focus on their multifaceted interactions with various immune cell populations. The present study discusses how CAFs utilize cytokine networks, metabolic reprogramming and immune checkpoint regulation to establish an immunosuppressive TME. Clinical translation should prioritize FAP-directed therapies alongside αPD-1 to concurrently target CAF-immune crosstalk and metabolic competition in the TME.

## 1 Introduction

Tumor immune escape represents one of the critical biological characteristics enabling the initiation, progression and metastasis of malignant tumors (1). Under normal physiological conditions, the body’s immune system identifies and eliminates abnormal cells through sophisticated multi-layered defense mechanisms, which is called “immune surveillance” (2). However, cancer cells have developed multiple complex mechanisms to evade recognition and attack by the immune system, a capability termed “immune escape” (3). From a molecular perspective, tumor immune escape primarily involves three key aspects: defective antigen presentation, formation of an immunosuppressive microenvironment, and activation of immune checkpoints (4). Cancer remains one of the leading causes of death worldwide, despite significant advancements in treatment modalities such as chemotherapy, radiation therapy and immunotherapy (5, 6). One of the major challenges in effectively treating cancer is immune evasion (7). Immune evasion not only facilitates tumor progression but also limits the efficacy of immunotherapies, which are designed to enhance the body’s natural defenses against cancer (8, 9).

The tumor microenvironment (TME) is a complex ecosystem comprising cancer cells, immune cells, and stromal cells such as cancer-associated fibroblasts (CAFs) (10, 11). CAFs are the most abundant stromal cells in the TME and have significant impacts on tumor growth, invasion and drug resistance (12). Recent studies have also shown that CAFs play a crucial role in maintaining the anti-tumor immune response (13). Through direct cell-cell interactions and paracrine signaling, CAFs modulate the function of various immune cells, including T cells, macrophages, and myeloid-derived suppressor cells (MDSCs), fostering an immunosuppressive milieu (14). This review discusses the key molecular mechanisms by which CAFs facilitate immune escape, which provides insights into potential therapeutic strategies to counteract CAF-mediated immunosuppression in cancer.

## 2 CAFs and their subtypes

CAFs are a diverse group of cells with distinct phenotypes and functions (15). CAFs lack a universal marker but are commonly identified by the expression of α-smooth muscle actin (α-SMA, encoded by *acta2*), fibroblast activation protein (FAP), platelet-derived growth factor receptors (PDGFRα/β), vimentin, and fibronectin (16). Based on transcriptomic and functional analyses, CAFs can be broadly categorized into several major subtypes (**Table 1**). Emerging spatial transcriptomic studies reveal that these subtypes exhibit distinct spatial niches within tumors, with FAP+ myCAFs predominantly localizing to collagen-rich invasive fronts while α-SMA+ iCAFs cluster near angiogenic vasculature, illustrating their specialized roles in stromal remodeling versus immune modulation (26).

**Table 1:** The subtypes of CAFs.

Subtype	Marker molecules	Key signaling pathways	Primary functions	Associated cancer types	Reference
Myofibroblastic CAFs (myCAF)	α-SMA, FAP and COL1A1	TGF-β/Smad and YAP1	Fibrosis promotion	Pancreatic cancer and breast cancer	(17, 18)
Inflammatory CAFs (iCAF)	IL-6, CXCL12 and LIF	JAK/STAT3 and NF-κB	Immunosuppression, inflammatory niche formation	Bladder cancer and breast cancer	(19, 20)
Antigen-presenting CAFs (apCAF)	MHC-II and CD74	IFN-γ/JAK1	Antigen-presenting cell mimicry	Gastric cancer and pancreatic cancer	(21, 22)
Metabolic CAFs (meCAF)	LDHA, MCT4 and GLUT1	HIF-1α and MYC	Metabolic reprogramming and nutrient competition	hepatocellular carcinoma and colorectal cancer	(23, 24)
Perivascular CAFs pvCAF	NG2, DES and RGS5	PDGF/Notch	Vascular remodeling and blood-tumor barrier formation	melanoma and pancreatic cancer	(25)

## 3 CAFs-Immune cells interactions and immune evasion

**Figure 1:**
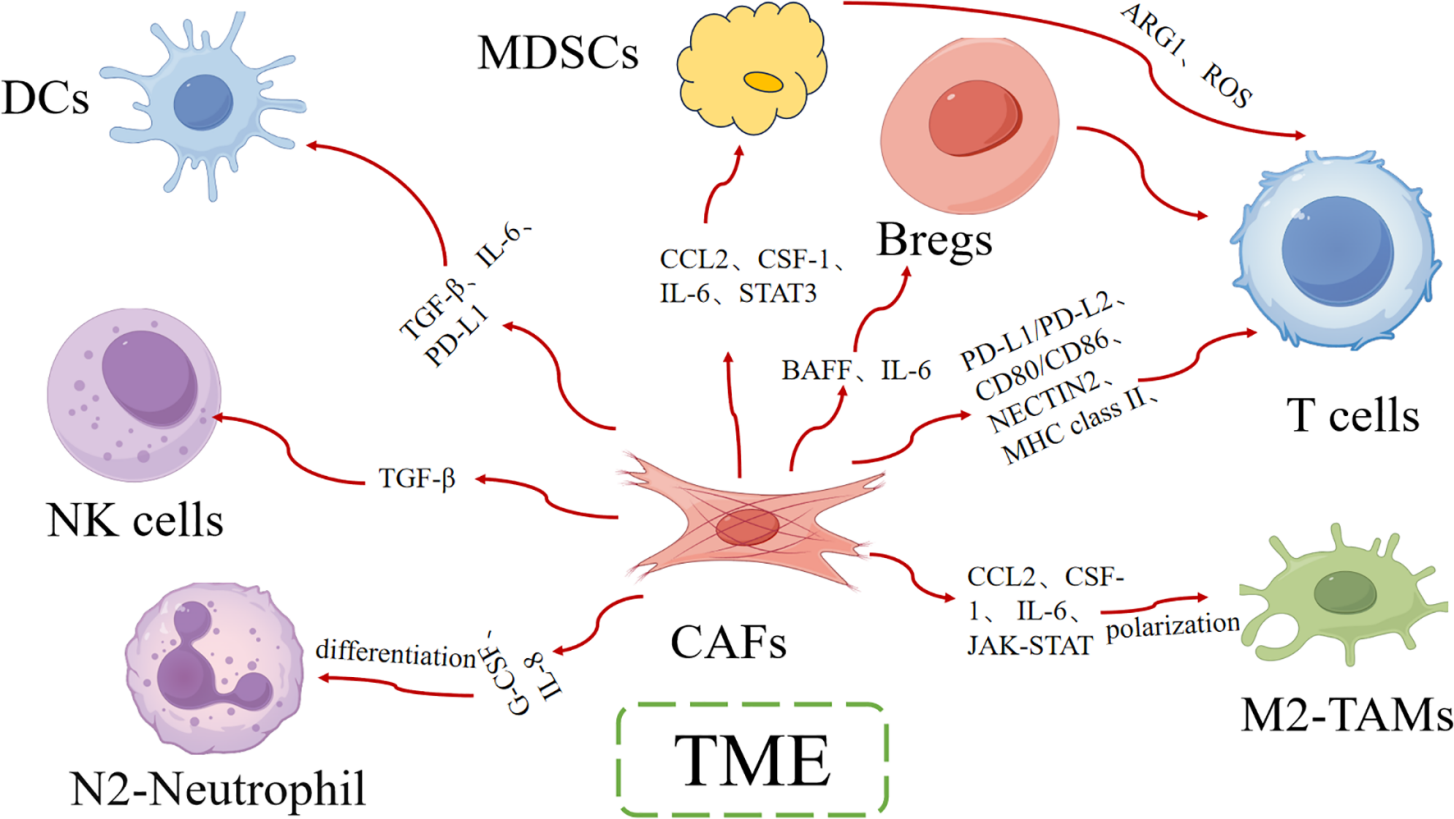
The interaction of CAFs with different immune cells.

Some studies suggest that CAFs dynamically regulated antitumor immunity through multifaceted interactions with immune cells, creating an immunosuppressive microenvironment that promotes therapy resistance (27). Understanding how CAFs interact with immune cells is essential for developing more effective cancer therapies (**Figure 1**).

### 3.1 CAFs and T cells

CAFs, as the predominant stromal cell component in the TME, shape a highly immunosuppressive microenvironment through multidimensional interactions with T cells, thereby promoting tumor immune evasion and therapeutic resistance (28, 29). These interactions involve multiple mechanisms, including direct cell-to-cell contact, secretion of soluble factors, metabolic reprogramming, and the formation of physical barriers (30, 31). Their complexity and dynamics have become a major focus in current cancer immunotherapy research.

CAFs directly regulate T cell functions. CAFs induce T cell exhaustion by upregulating co-inhibitory molecules such as PD-L1/PD-L2 and CD80/CD86, which bind to receptors including PD-1 and CTLA-4 on T cell surfaces (32–34). A single-cell sequencing study reveal that approximately 40% of CAFs in pancreatic cancer highly express PD-L2, which exhibits 30% higher binding affinity to PD-1 compared to PD-L1 (35). Recent mechanistic studies using 3D CAF-T cell coculture systems have demonstrated that CAF-derived NECTIN2 directly engages CD226 on CD8^+^ T cells, inducing caspase-3-dependent apoptosis. CRISPR-mediated knockout of NECTIN2 in pancreatic CAFs reduced T cell apoptosis by 67% *in vitro (* 13). Flow cytometry analysis revealed that TNBC-derived CAFs exhibit 3.2-fold higher surface NECTIN2 expression compared to normal mammary fibroblasts (p<0.001), suggesting tumor-specific regulation of this pathway. Building upon these established mechanisms, the novel single-cell RNA-seq data identified IGFBP5 as a key upstream regulator of NECTIN2 expression in metastatic CAF subsets (26). Furthermore, certain CAF subsets, such as apCAFs, aberrantly express MHC class II molecules, driving CD4^+^ T cell differentiation toward regulatory T cells rather than effector T cells (21, 26). In the lung cancer microenvironment, this “pseudo-antigen presentation” can increase regulatory T cell (Treg) proportions by 3- to 5-fold (21).

CAFs further suppress T cell function through soluble factor-mediated paracrine signaling mechanisms. As a master regulator of immune homeostasis, transforming growth factor-beta (TGF-β) plays a multifaceted role in tumor immunology, with CAFs constituting the predominant cellular source of this cytokine in the TME (36–39). TGF-β signaling induces profound dysfunction in CD8^+^ cytotoxic T lymphocytes through inducing the epigenetic silencing of effector molecules, the transcriptional downregulation of EOMES and TBX21 (the critical transcription factors for cytotoxic differentiation) and the inhibition of mitochondrial oxidative phosphorylation (40–43).

In addition to the aforementioned mechanisms, CAFs can also influence T cell-mediated immune responses by affecting T cell metabolism and remodeling the ECM. For instance, CAFs significantly reduce the glucose concentration in the TME to one-tenth of that in normal tissues by overexpressing glucose transporter 1 and hexokinase 2, which notably decreased the proliferation of T cells and inhibited the mTOR signaling pathway within these cells (44). Moreover, CAFs reshape the ECM through the secretion of matrix-degrading enzymes, leading to a dense fibrotic stroma that physically impedes T cell infiltration and interaction with tumor cells (45). Altogether, these multifaceted interactions highlight the complex role of CAFs in modulating the tumor immune landscape and underscore the need for targeted strategies to disrupt their immunosuppressive functions.

### 3.2 CAFs and tumor-associated macrophages

Beyond their multifaceted immunosuppressive effects on T cells, CAFs also establish critical crosstalk with tumor-associated macrophages (TAMs), forming another key immunosuppressive axis in the tumor microenvironment. The crosstalk between CAFs and TAMs constitutes a critical axis in tumor immune evasion, creating a profoundly immunosuppressive microenvironment (46). CAFs secrete key chemokines and cytokines, including CCL2, CSF-1 and IL-6, which promotes monocyte egress from peripheral blood, resulting in a 3-5-fold enhancement of TAM infiltration within the tumor microenvironment (47–49). The CAF-derived factors induce profound phenotypic changes in recruited macrophages in hepatocellular carcinoma through JAK-STAT pathway activation (50). This signaling cascade drives macrophage polarization toward an immunosuppressive phenotype (51). Notably, this paracrine signaling axis also upregulates immune checkpoint molecule expression, with CAF-conditioned media inducing a great increase in PD-L1 surface expression on TAMs as measured by flow cytometry. Mechanistic studies reveal that this effect is mediated through both STAT3-dependent transcriptional activation and post-translational stabilization of PD-L1 protein (52).

The biological consequences of this CAF-TAM crosstalk include: enhanced phagocytic resistance of tumor cells, increased production of immunosuppressive IL-10, impaired antigen presentation capacity and promotion of angiogenesis through VEGF secretion (22, 53–55). In the clinical context, the alliance of CAF-TAM correlates with several adverse outcomes. Specifically, in liver cancer, the interaction between TAMs and CAFs is crucial in shaping the immune barrier, and the disruption of this communication potentially enhance immunotherapy effectiveness and improve clinical outcomes of patients (56). In conclusion, this interaction between CAFs and TAMs is a key mechanism of immune evasion in the TME.

### 3.3 CAFs and myeloid-derived suppressor cells

Building upon their interactions with TAMs, CAFs also establish critical immunosuppressive networks with myeloid-derived suppressor cells (MDSCs), further reinforcing the immune-evasive nature of the tumor microenvironment. MDSCs are a heterogeneous population of myeloid cells at various stages of differentiation, with immunosuppressive activity as the main feature (57). MDSCs can promote immune evasion and tolerance by recruiting Treg via CD40-CD40 ligand signaling (58). Besides, indoleamine 2,3-dioxygenase (IDO) or arginase 1 (ARG1) expressed by MDSCs contribute to the immunosuppressive metabolic microenvironment (59, 60). In the TME, MDSCs and CAFs are two key immune-suppressive cells that promote tumor immune evasion and progression through complex interactions (61). CAFs recruit MDSCs from the bone marrow to the tumor site by secreting cytokines such as CCL2, CSF-1 and IL-6. These cytokines also can activate MDSCs, thereby enhancing their ability to suppress T cell function (62). A recent study found that cytokines by CAFs enhanced the immune-suppressive capabilities of MDSCs and further secreting ARG1 and reactive ROS to inhibit T cell proliferation and activity (63). CAFs and MDSCs jointly deplete arginine and tryptophan in the tumor microenvironment, leading to metabolic dysfunction in T cells (64). This metabolic competition deprives T cells of essential nutrients, inhibiting their proliferation and function. Additionally, the ROS produced by MDSCs further damage the T cell receptor (TCR), reducing T cell reactivity to tumor antigens (65). CAFs and MDSCs also have a synergistic effect in immune suppression (66). CAFs reshape the ECM to form a physical barrier that restricts immune cell infiltration. MDSCs further suppress T cell function by secreting immune-suppressive molecules such as TGF-β and IL-10 (67). This synergistic effect makes immune suppression in the tumor microenvironment more pronounced, thereby promoting tumor progression and resistance to treatment.

### 3.4 CAFs and dendritic cells

Beyond their interactions with MDSCs, CAFs also engage in complex crosstalk with dendritic cells (DCs), another crucial component of the tumor immune microenvironment. One of the key interactions that contribute to immune evasion involves the relationship between CAFs and DCs. This interaction is complex and multifaceted, influencing both the function and phenotype of DCs, thereby facilitating tumor progression and resistance to immune surveillance (67). CAFs significantly impact the maturation and function of DCs. In a study involving the fusion of DCs and CAFs, it was observed that DC/CAF fusion cells expressed higher levels of co-stimulatory molecules such as CD80, CD86 and MHC II compared to immature DCs, suggesting that CAFs influence the maturation state of DCs, potentially skewing their function towards a more immunosuppressive phenotype (68). CAFs also impair the antigen-presenting function of DCs, which is crucial for initiating T cell-mediated immune responses. For instance, CAFs secrete factors such as PGE2 and TGF-β, which downregulate the expression of molecules important for antigen presentation, including MHC class II in DCs (28). This impairment in DC function reduces the ability of activation of CD4^+^ and CD8^+^ T cells, thereby facilitating immune evasion. Additionally, CAFs also influence the recruitment of DCs to the tumor site. By secreting chemokines such as CXCL12, CAFs attract DCs into the TME (69, 70). Emerging evidence suggests that while CAFs actively recruit DCs into the TME, these DCs often exhibit functional impairment due to CAF-mediated immunosuppression. This creates a paradoxical scenario where DCs are physically present but functionally compromised, ultimately failing to initiate proper T cell activation and contributing significantly to tumor immune evasion (71).

### 3.5 CAFs and natural killer cells

Complementing their suppressive effects on adaptive immune cells, CAFs also exert profound inhibitory effects on innate immunity, particularly by impairing the cytotoxic function of natural killer cells (NKCs). NKCs are innate immune cells that can recognize and kill tumor cells without prior sensitization. However, the presence of CAFs significantly impair the function of NKCs, contributing to immune evasion (72). Cytokines and chemokines produced by CAFs which are shown to suppress the cytotoxic activity of NKCs (72). These cytokines also induce the expression of inhibitory receptors on NKCs, further dampening their ability to recognize and kill tumor cells (72). CAFs deplete essential nutrients in the TME, creating a hostile environment for NKCs. Malchiodi et al. point out that CAFs consume a large of arginine and tryptophan, which are critical for NKCs activation and function (72). CAFs also interact directly with NKCs through cell surface molecules, leading to the downregulation of activating receptors on NKCs (71). This direct contact can inhibit NKCs cytotoxicity and promote immune tolerance within the TME. In summary, inhibiting the secretion of immunosuppressive cytokines by CAFs or blocking the direct cell-to-cell contact between CAFs and NKCs restore NKCs function and improve anti-tumor immunity (73).

### 3.6 CAFs and B cells

Extending their immunosuppressive influence beyond innate and T cell immunity, CAFs also engage in critical interactions with B cells, further shaping the tumor immune landscape. The interactions between CAFs and B cells are multifaceted and can influence both the function and phenotype of B cells within the TME. Recent studies have shown that CAFs modulate the immune landscape by interacting with various immune cells, including B cells (74). CAFs secrete a variety of cytokines to inhibit B cell activation, proliferation and antibody production, as well as reducing the overall immune response (75). Regulatory B cells (Bregs) is a subpopulation which are known for their immunosuppressive properties (74). CAFs utilizes at least two pathways to interact with Bregs: (1) secreting TGF-β to promote the differentiation of B cells into Bregs (74), and (2) secreting CXCL13 to recruit mature Bregs into TME (76, 77). In melanoma and colorectal cancer models, Bregs recruited by CAFs directly impairing cytotoxic T cell responses and contributing to anti-PD-1 therapy resistance (76, 77). All in all, understanding these interactions and developing strategies to disrupt them could significantly enhance the efficacy of cancer immunotherapies improve patient outcomes.

### 3.7 CAFs and neutrophils

Completing the spectrum of their immunomodulatory effects, CAFs additionally orchestrate critical interactions with tumor-associated neutrophils (TANs), establishing yet another immunosuppressive axis in the tumor microenvironment. The bidirectional communication between CAFs and tumor-associated neutrophils has emerged as a critical axis in facilitating immune evasion. CAFs serve as the primary architects of neutrophil recruitment within tumors, secreting an array of cytokines and chemokines. The most potent of these is IL-8 (22). Subsequently, these recruited neutrophils polarize into an pro-tumor phenotype, characterized by significantly increased ARG1 expression, elevated PD-L1 surface levels and enhanced MMP-9 secretion, collectively establishing a profoundly immunosuppressive microenvironment (36, 78, 79). The central role of CAF and neutrophils’ interaction in creating and maintaining an immunosuppressive tumor microenvironment is an attractive target for cancer therapy.

## 4 Molecular mechanisms of CAFs-mediated immune evasion

**Figure 2:**
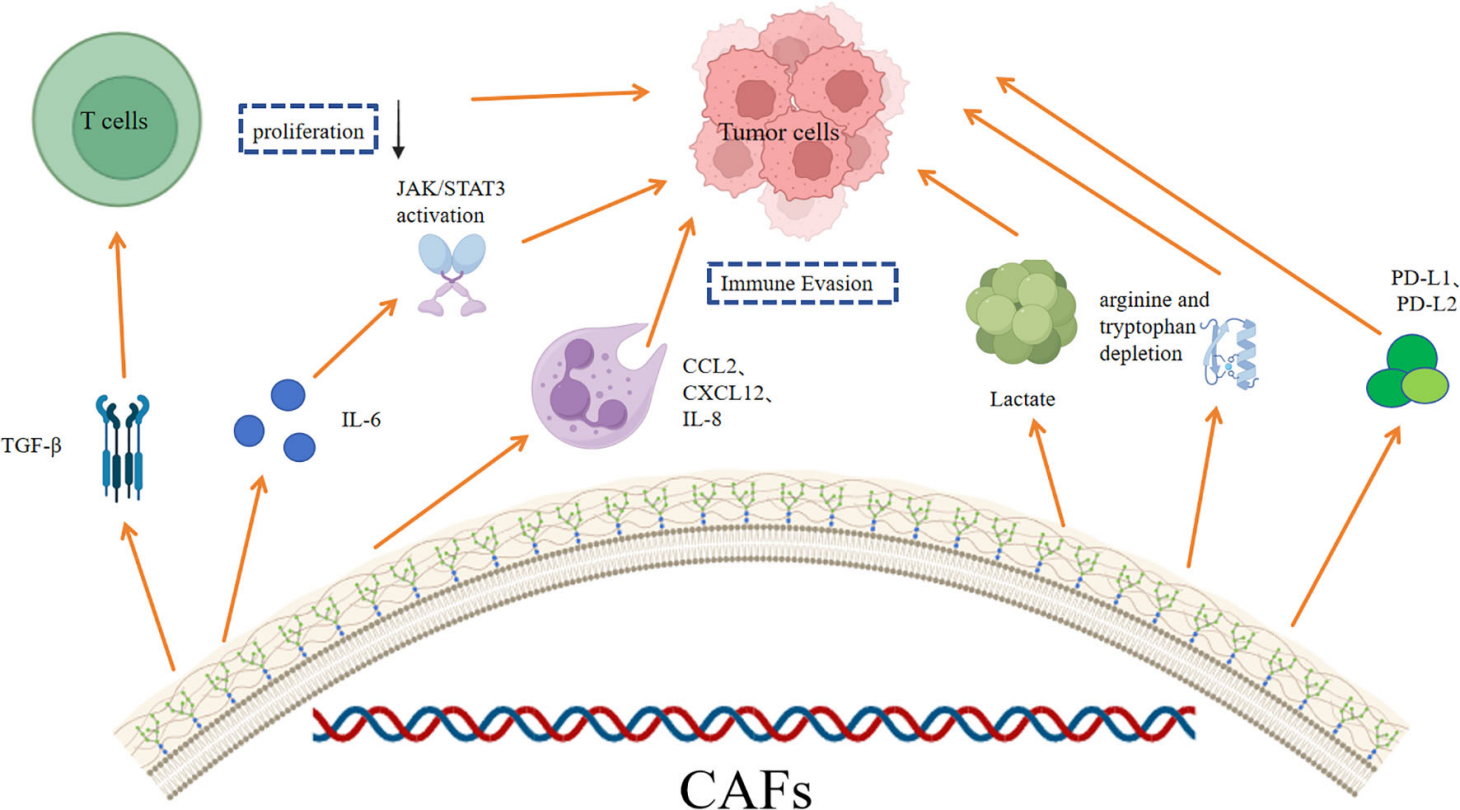
Several major CAFs-mediated immune escape mechanisms.

CAFs play a crucial role in the immune evasion through various mechanisms (**Figure 2**). Understanding the molecular mechanisms of CAFs-mediated immune evasion has important implications for cancer therapy.

### 4.1 Cytokine-mediated immunosuppression

CAFs modulate the immune microenvironment through the secretion of various cytokines and chemokines, which influence the recruitment, activation, and function of immune cells within the TME (80).

TGF-β is a key cytokine secreted by CAFs that has potent immunosuppressive effects. TGF-β can inhibit the proliferation and function of T cells, promote the differentiation of regulatory T cells, and enhance the immunosuppressive phenotype of other immune cells such as macrophages and dendritic cells (81). Additionally, TGF-β signaling can induce the expression of immune checkpoint molecules like PD-L1 on tumor cells and stromal cells, further contributing to immune evasion (82). The TGF-β signaling axis is thus a critical pathway through which CAFs mediate immune suppression in the TME.

IL-6 is another important cytokine produced by CAFs that drives immune suppression via the JAK/STAT3 signaling pathway. IL-6 activates the JAK/STAT3 pathway in immune cells, leading to the upregulation of anti-inflammatory and immunosuppressive genes (83). This pathway also promotes the differentiation of Tregs, inhibits the function of cytotoxic T cells and enhances the production of immunosuppressive cytokines such as IL-10 (8). Moreover, the IL-6/JAK/STAT3 pathway influence the function of myeloid cells, including macrophages and neutrophils, skewing them towards a more immunosuppressive phenotype (84). Targeting the IL-6/JAK/STAT3 pathway has emerged as a promising strategy to overcome CAF-mediated immune suppression in cancer.

CAFs secrete a variety of chemokines, such as CCL2, CXCL12, and IL-8 playing crucial roles in the recruitment and positioning of immune cells. For example, CCL2 attracts monocytes and macrophages, while CXCL12 is involved in the recruitment of T cells and dendritic cells (85). Additionally, these chemokines influence the phenotype and function of immune cells, further contributing to immune evasion (86).

### 4.2 Metabolic reprogramming of TME

Lactate is a byproduct of glycolysis and is often produced in high amounts by cancer cells and CAFs (87). The accumulation of lactate in the TME leads to acidification, creating a low pH environment that is immunosuppressive. This acidic environment also suppresses the function of T cells and other immune cells, thereby facilitating immune evasion (88). Additionally, lactate can activate the Wnt/β-catenin signaling pathway in tumor cells, further promoting their energy metabolism and proliferation (89).

CAFs deplete essential amino acids in the TME, such as arginine and tryptophan, through increased metabolic activity. This depletion can impair the function of immune cells, particularly T cells, which require these amino acids for activation and proliferation (90). Recent research showed that CAFs express high levels of ARG1, an enzyme that degrades arginine, leading to a deficiency of this amino acid in the TME (91). Similarly, the expression of tryptophan-degrading enzymes like indoleamine-2,3-dioxygenase by CAFs lead to tryptophan depletion, further suppressing T cell function (92). This metabolic competition between CAFs and immune cells creates an immunosuppressive environment that promotes tumor progression.

CAFs exhibit significant alterations in lipid metabolism, which can impact the TME and immune cell function. High expression of fatty acid synthase, carnitine palmitoyl transferase 1 and CD36 in CAFs promotes fatty acid oxidation and the accumulation of intracellular lipid droplets (90). These metabolic changes can lead to the exhaustion of cytotoxic T lymphocytes and provide fuel for tumor cell fatty acid metabolism, contributing to tumor proliferation and metastasis (93, 94). Additionally, the altered lipid metabolism in CAFs affect the function of other immune cells, further contributing to immune suppression (90).

Hypoxia is a common feature of the TME and induce significant metabolic changes in CAFs. Under hypoxic conditions, CAFs upregulate the expression of hypoxia-inducible factor, which drives the expression of genes involved in glycolysis and other metabolic pathways (95). This metabolic reprogramming allows CAFs to adapt to the low-oxygen environment and further supports the growth and survival of tumor cells. Hypoxia also enhances the secretion of immunosuppressive cytokines and chemokines by CAFs, such as TGF-β and IL-6, which inhibit the function of immune cells and promote immune evasion (96). Moreover, hypoxia-induced metabolic changes in CAFs can lead to the production of reactive oxygen species (ROS), which can damage immune cells and further suppress their function (97).

### 4.3 Immune checkpoint regulation

CAFs significantly influence the expression of immune checkpoint molecules such as PD-L1 and PD-L2 within the TME (98). Studies have shown that IFN-γ can significantly upregulate the expression of PD-L1 and PD-L2 in various tumor cell lines, thereby enhancing immune evasion (99). The upregulation of PD-L1 and PD-L2 on CAFs and other cells in the TME contributes to the suppression of T cell-mediated immune responses, facilitating tumor progression (100).

In addition to the well-known PD-1/PD-L1 axis, CAFs also modulate the expression of other non-canonical immune checkpoints, such as VISTA and LAG-3. VISTA (V-domain Ig suppressor of T cell activation) is an inhibitory receptor that can suppress T cell activation and proliferation. Similarly, LAG-3 (lymphocyte-activation gene 3) is another immune checkpoint molecule that can inhibit T cell function and contribute to immune exhaustion (101). The expression of these non-canonical checkpoints by CAFs or other cells in the TME can further dampen the immune response, creating a more immunosuppressive environment. Targeting these non-canonical checkpoints, in combination with PD-1/PD-L1 inhibitors, may provide a more comprehensive approach to overcoming immune evasion in cancer.

Fas/FasL is a critical death receptor-ligand system that mediates apoptosis signaling, which is another pathway affected by CAFs in immune evasion (102). Fas ligand is expressed by various cells in the TME, including CAFs, and can induce apoptosis in Fas-expressing T cells. This interaction leads to the elimination of activated T cells, thereby reducing the overall immune response against the tumor (101). The expression of FasL by CAFs can create a local environment that is hostile to T cells, promoting immune evasion and tumor progression. Targeting the Fas/FasL pathway may help to preserve the function of T cells and enhance anti-tumor immunity. At the same time, emerging clinical evidence supports targeted CAF immune crosstalk, and ongoing trials of FAP/PD-L1 dual targeted CAR-T cell (NCT04328026) and TGF-β/PD-1 combination therapy (NCT0366871) have shown a 36-41% improvement in response to treatment resistant tumors (57). CAFs modulate the immune microenvironment through the CD73/adenosine pathway (103). CD73 is an ectoenzyme that converts extracellular AMP into adenosine, a potent immunosuppressive molecule (104). High levels of adenosine in the TME inhibit the function of T cells and other immune cells, promoting immune evasion. CAFs express high levels of CD73, contributing to the accumulation of adenosine in the TME (101, 105). This pathway is targeted by inhibitors of CD73 or adenosine receptors, potentially enhancing the efficacy of cancer immunotherapies.

## 5 Conclusion

Cancer associated fibroblasts coordinate immune suppression through three core mechanisms: cytokine network (TGF-β/IL-6), metabolic symbiosis (lactate/arginine consumption), and immune checkpoint crosstalk (PD-L1/CD73). These pathways drive T cell dysfunction, macrophage polarization, and bone marrow suppression, forming a therapeutic barrier. Emerging strategies such as JAK/STAT inhibitors, metabolic disruptors, and adenosine blockade have shown promise in epithelial cancers where CAF immune interactions dominate, in stark contrast to the limited role of the matrix in hematopoietic malignancies (94). Space single-cell localization reveals tissue-specific CAF heterogeneity, guiding precise treatment that combines matrix reprogramming with immunotherapy. Future translation requires CAF derived extracellular vesicle biomarkers for patient stratification and targeted delivery systems to avoid drug resistance.
